# Planning of births and childhood undernutrition in Nepal: evidence from a 2016 national survey

**DOI:** 10.1186/s12889-020-09915-8

**Published:** 2020-11-25

**Authors:** Ishwar Tiwari, Kiran Acharya, Yuba Raj Paudel, Bhim Prasad Sapkota, Ramesh Babu Kafle

**Affiliations:** 1 Health for Life, RTI International, Kathmandu, Nepal; 2 New ERA, Rudramatimarg, Kalopul, Kathmandu, Nepal; 3 School of Public Health, University of Alberta, Edmonton, Canada; 4 Ministry of Health and Population, Ram Shah Path, Kathmandu, Nepal; 5 Center for International Health, Ludwig Maximilian University of Munich (CIH-LMU), Munich, Germany; 6 Centre of Population and Development, Purbanchal University, Munich, Nepal

**Keywords:** Planning of birth, Undernutrition, Stunting, Underweight, Nepal

## Abstract

**Background:**

Childhood undernutrition is a significant public health issue in low-and middle-income countries, including Nepal. However, there is limited evidence showing the association between the planning of birth (PoB) and childhood undernutrition (stunting and underweight). We aimed to investigate the relationship between PoB and childhood undernutrition in the current study.

**Methods:**

We used the Nepal Demographic and Health Survey (NDHS) 2016 data, a nationally representative cross-sectional household survey. We used two anthropometric indicators of childhood undernutrition as the outcome of this study. PoB is the main predictor. We used binary logistic regression with sampling weights to estimate adjusted odds ratios (ORs) and 95% confidence intervals (CIs) to examine the association between the PoB and childhood undernutrition. Unless stated, the significant association between the variables is calculated with *p* < 0.001.

**Results:**

The overall prevalence of stunting was 35.8%, and underweight was 27.1% in children under 5 years of age in Nepal. We found a higher rate of stunting (52.7%) and underweight (41.1%) in children with birth order > 3 and < 2 years of the interval between birth and subsequent birth (IBBSB). The association between the children’s birth order and the prevalence of undernutrition had strong statistical significance. Mother’s age at marriage (*p* = 0.001), underweight mother, mother’s education, father’s education, wealth quintile, no exposure to mass media, children’s age, and place of residence(*p* = 0.001) were significantly associated with childhood undernutrition. The result of the multiple logistic regression showed that children with birth order one and 12–24 months of the interval between marriage and first birth (IBMFB) had significantly decreased odds of stunting than those children with birth order one and < 12 months of IBMFB (OR 0.6, 95% CI 0.4–0.9).

**Conclusion:**

The findings of the study demonstrate that PoB has a protective effect on childhood undernutrition. Delaying of childbirth until 12–24 months after marriage was found to be associated with reduced childhood stunting odds. To mitigate childhood undernutrition, Nepal’s government needs to promote delayed childbearing after marriage while focusing on uplifting the household economics status and wide coverage of and utilization of mass media.

## Background

Childhood undernutrition is a significant public health issue globally. It is estimated that among children under 5 years of age, one-third are stunted (178 million), and 112 million are underweight [1]. Childhood undernutrition accounts for more than half of the global deaths in children younger than 5 years of age [2, 3]. It causes a substantial increase in overall disease burden, particularly in low-and middle-income countries, including Nepal [2, 3].

The government of Nepal (GoN) has made a clear commitment to addressing undernutrition, including childhood undernutrition in Nepal [4]. Nepal signed up to the global Scaling Up Nutrition (SUN) movement in 2011, agreed upon by the World Health Assembly (WHA) in 2012 [5]. The SUN movement aimed at a 40% reduction in global stunting among children under 5 years of age by 2025 [6]. Currently, Nepal’s annual average rate of reduction (AARR) of stunting is 0.031, but it needs to maintain an AARR of 0.043 to achieve the global target of stunting in children under 5 years of age [7]. Nepal endorsed a multi-sector approach for nutrition and prioritized improving child nutrition in its five-year Multi-sector Nutrition Plan (MSNP) 2013–2017 [8]. The Government of Nepal is committed to the declaration of the United Nations (UN) General Assembly’ 2016–2025 period as the decade of action on nutrition’ [9]. In 2017, Nepal endorsed MSNP 2018–2022 and renewed its commitment to nutrition [7]. Nepal also supports internationally agreed targets; specifically, the Sustainable Development Goal (SDG) target 2.2 on ending all forms of malnutrition by 2030 [10, 11]. SDG 2 targets to reduce the prevalence of undernourishment to 3% and underweight prevalence to 5% in children under 5 years of age by 2030 [5]. All the national nutritional targets of Nepal are also aligned with the Global Nutrition Targets [4].

Department of health service (DoHS), under the Ministry of Health and Population (MoHP), Nepal, executes several national-level nutrition programs, such as Vitamin A supplementation, growth monitoring, Infant and young child feeding (IYCF), iron supplementation, iodine fortification. Under the common framework of MSNP, the GoN has also initiated several large-scale, multi-sector, integrated nutrition projects and programs, with support from its development partners. For example, Nepal started United States Agency for International Development (USAID) supported *SUAAHARA* (Good nutrition) projects, KISAN (Knowledge-based Integrated Sustainable Agriculture in Nepal), and SABAL (Sustainable Action for Resilience and Food Security); World Bank-supported *Sunaulo Hazar din* (Golden 1000 days); European Union and the United Nations International Children Emergency Fund (UNICEF) supported Maternal and Young Child Nutrition Security Initiative in Asia(2011–2015) and *Poshan ko Lagi Hatemalo* (Partnership for Improved Nutrition) (2016–2019) [4]. Such an aggressive implementation of the nutrition-specific program has contributed to a progressive decline in the prevalence of undernutrition in children under 5 years of age in Nepal. In 2006, stunting was was 49%, which decreased to 41% in 2011, and 36% in 2016 [12–14]. Underweight also reduced from 39% in 2006 to 29% in 2011 and 27% in 2016 [12–14]. However, Nepal has still a considerable prevalence rate of stunting and underweight in children under 5 years of age.

Family planning has a positive influence on child health and nutritional outcomes [15]. It allows a couple to plan the timing of their childbirth, the number of deliveries, and the childbirth spacing [15, 16].

Previous studies have shown that shorter birth spacing and frequent childbearing cause adverse effects not only on maternal and child health but also lead to poor nutritional outcomes [15, 17–19]. Childbearing at a younger age also causes poor nourishment of children [20]. Therefore, some international agencies have advocated empowering women to partake in family planning, which also supports to improve maternal, infant, and young child nutrition [20]. For example, *SUAAHARA* (Good Nutrition) program in Nepal advocates for women empowerment and has integrated family planning as one of the eight essential nutrition actions to promote maternal and child nutrition [21, 22]. The theory of change of MSNP-II 2018–2022 has also accepted family planning and reproductive health services to achieve nutritional targets under the health sector response [7]. Although family planning may contribute to better nutrition outcomes in under-five children, it has received little attention in low-and middle-income countries [23]. Only a few global studies exist which have investigated the relationship between family planning and childhood undernutrition [15, 20, 23–25]. Nepal’s National Nutrition Policy and Strategy 2004 (revised in 2008) and National Health Policy 2019 are the leading policy documents guiding nutritional interventions in the health sectors. Still, both documents are unaware of the relationship between PoB and nutritional outcomes of children due to the lack of stringent evidence. Therefore, this paper aims to examine the association between PoB and undernutrition in children under 5 years of age in Nepal.

## Methods

### Data source

We used data from the NDHS conducted in 2016. NDHS is a nationally representative cross-sectional household survey conducted every 5 years. For the 2016 NDHS, the survey sample of 12,862 is representative at the national and provincial levels, for ecological zones and development regions, and the urban and rural areas [14]. The 2016 NDHS used the sampling frame from the 2011 National Population and Housing Census (NPHC), which was conducted by the Central Bureau of Statistics (CBS). The details of the sampling procedure are available elsewhere [14]. The survey collected a broad range of socioeconomic, demographic, and health data from all eligible men and women of age group 15–49 by administering six well-structured and standardized questionnaires (the household questionnaire, the woman’s questionnaire, the man’s questionnaire, the biomarker questionnaire, the fieldworker questionnaire, and the verbal autopsy questionnaire for neonatal death). All questionnaires were first finalized in English and then translated into Nepali, Maithili, and Bhojpuri. ICF Institutional Review Board (IRB) reviewed the survey. The survey also received ethical approval from the Nepal Health Research Council (NHRC). All interviewers underwent 2 weeks of a training course using a standard protocol manual, followed by 1 week of Computer Assisted Personal Interview (CAPI) training before data collection [26]. Written consent was sought from each participant following the NHRC guidelines. The 2016 NDHS yielded a response rate of 99%.

### Definition of variables

We considered two common anthropometric indicators of undernutrition, namely stunting (low height-for-age) and underweight (low weight-for-age), as the outcome variables for this study. As per the World Health Organization (WHO) criteria, children with less than negative two (<− 2) standard deviation.

(SD) below the mean are considered as stunted and underweight [27]. We used normalized z-scores in the NDHS dataset for height-for-age and weight-for-age.

For this study, the main predictor of interest is PoB, which is composed of the intersectional axes of birth order, the interval between marriage and first birth (IBMFB), and the interval between births and subsequent births (IBBSB). We categorized the birth order into three categories: one, 2–3, and > 3. We grouped IBMFB into < 12 months, 12–24 months, 25–36 months, and more than 36 months for the first order birth. For birth order 2–3 and > 3, we divided IBBSB into ≤24 months and > 24 months. In total, we created eight intersectional axes of the PoB.

### Selection of study sample

**Fig. 1:**
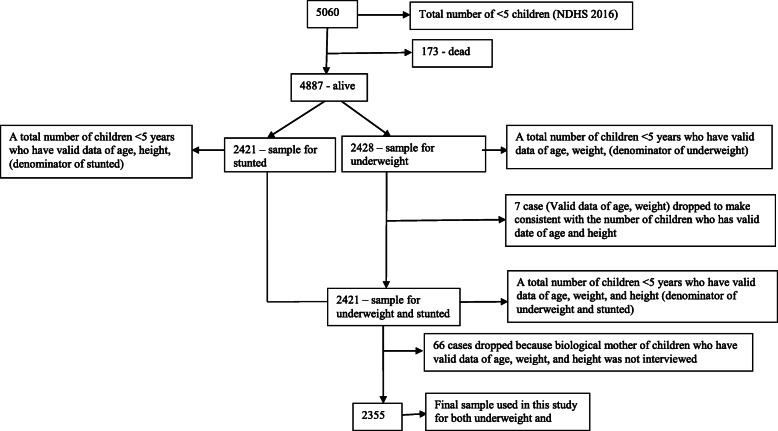
Flowchart of the study sample

The survey identified 5060 children under 5 years of age, of which 4887 were alive and 173 were dead. After excluding missing cases whose valid date of birth and valid measurement of both height and weight were not reported, NDHS reported 2421 stunted children and 2428 underweight children. The difference between the stunting and underweight sample size was due to the difference of flagged/missing cases in height and weight measurements. We dropped seven denominator cases used in low weight-for-age, i.e., underweight, to make it consistent with the total number of children, i.e., the denominator used in stunted (Fig. 1). We also excluded the child whose biological mother was not interviewed. Thus, our final study sample included 2355 children (for both stunting and underweight) for the analysis based on the inclusion criteria: children who have valid data of age, weight, height, and whose biological mother was interviewed.

### Statistical analysis

We used STATA version 15.0 to conduct data analyses. Descriptive analysis of the outcome and covariates are shown in the table. We performed a chi-square test to assess the association between the predictors and outcome variables. We conducted a binary logistic regression to examine the association between PoB and childhood undernutrition. We controlled for the potential confounding variables in the analysis, such as mother’s age at marriage in years, mother’s current age in years, mother’s BMI, mother’s anemia, caste, mother’s education, father’s education, place of residence, mother’s occupation, wealth quintile, exposure to mass media, sex of the children, age of the children in months, place of residence, ecological region, province, and household food security. Adjusted ORs and 95% CIs were calculated to show the effect and significance of the association. We checked the collinearity effect among the predictors and investigated the potential interaction of the PoB with maternal nutrition and wealth quintile. We considered a *p*-value < 0.05 and 95% CIs for the significant relationship between the exposure and outcome variables. We presented the weighted figures to adjust for variations in the selection probabilities and interviews among participants. We also utilized the “Svy” command to account for complex survey design and provide unbiased estimates.

## Results

### Descriptive results

Table 1 presents the descriptive characteristics of the study population. The frequency distribution table shows the highest percentage of order 2–3 children (45.7%), with 35.1% of them having more than 24 months of the interval between birth and subsequent birth (IBBSB). 64.1% of the mothers were married at the age of 15 to 19 years, with a mean age at marriage of 17.7 years. Approximately two-thirds of the mothers (65.4%) had normal basal metabolic index (BMI), and more than half of the mothers (54.2%) of the children were not anemic. A considerable proportion of mothers who participated in the study were illiterate (34.6%), and 45.3% of the mothers had at least secondary education. Almost half of the mothers (46.2%) lived on agriculture. The male children’s share was slightly higher (52.2%) than female children (47.8%) in the survey.

**Table 1:** Descriptive statistics of the study population

Variables	Percentage	Frequency (***n*** = 2355)
**Planning of births**^a^		
Order 1 & < 12 month of IBMFB	9.2	216
Order 1 & 12–24 months of IBMFB	15.4	362
Order 1 & 25–36 months of IBMFB	7.5	176
Order 1 & > 36 months of IBMFB	5.7	134
Order 2–3 & < =24 months of IBBSB	10.6	249
Order 2–3 & > 24 months of IBBSB	35.1	825
Order > 3 & < =24 months of IBBSB	4.2	100
Order > 3 & > 24 months of IBBSB	12.2	285
**Mother’s age at marriage in years**		
< 15	12.6	297
15–19	64.1	1509
20–24	19.6	460
25–40	3.7	87
Mean age (sd) = 17.7 (3.3)		
**Mother’s current age in years**		
15–19	8.3	195
20–24	34.2	804
25–29	32.6	767
30–34	15.7	369
35–49	9.3	220
Mean age (sd) = 26.3 (5.7)		
**Mother’s BMI**^a^		
Normal	19.2	450
Underweight	65.4	1537
Obese	15.5	364
**Mother’s anemia**^a^		
Not anemic	54.2	1269
Anemic	45.8	1071
**Caste**		
Brahmin/Chettri	26.8	632
Terai/Madhesi other	20.2	476
Dalit	14.4	339
Newar/Janajatis	31.1	732
Muslim/other	7.5	176
**Mother’s education**		
Illiterate	34.6	815
Primary	20	472
Secondary	31.8	749
Higher	13.5	319
**Father’s education**^a^		
Illiterate	15.5	361
Primary	22.9	534
Secondary	44.2	1033
Higher	17.5	408
**Mother’s occupation**		
Not working	39.7	935
Agriculture	46.2	1089
Paid jobs	14.1	331
**Wealth quintile**		
Poorest	20.6	486
Poorer	21.8	513
Middle	22.7	535
Richer	21.6	510
Richest	13.2	311
**Exposure to mass media (newspaper/radio/television)**		
Not at all	23.1	545
Less than once a week	47.5	1118
At least once a week	29.4	692
**Sex of the children**		
Male	52.3	1231
Female	47.7	1124
**Age of the children**		
0–11 months	19.8	465
12–23 months	21.6	508
24–35 months	18.9	446
36–47 months	20.2	476
48–59 months	19.5	460
Mean age(sd) = 29.4 (17.2)		
**Place of residence**		
Urban	52.6	1239
Rural	47.4	1117
**Ecological region**		
Mountain	7.0	164
Hill	36.6	861
Terai	56.5	1330
**Province**		
Province 1	16.1	379
Province 2	27.4	645
Province 3	14.8	349
Province 4	7.7	182
Province 5	18.9	445
Province 6	6.4	151
Province 7	8.6	204
**Household food security**		
Food secure	41	965
Mild food insecure	22.7	535
Moderate food insecure	25.8	609
Severe food insecure	10.5	247

^a^number of cases missed from the variable:

Planning of birth- 8

Mother’s BMI- 4

Mother’s anemia- 15

Father’s education- 19

The representation of the study population by area of residence was quite similar (urban-52.6% and rural- 47.4%). Newar and Janajatis represented the largest percentage (31.1%) of the study participants, while Muslims/others had the least representation (7.5%) in the present study. The distribution of the study population was nearly uniform, around 21% for all categories of wealth quintile except the richest category, which was as low as 13.2%. Most children represented Province 2 (27.4%), while Province 6 had the least percentage of children who participated in the study. A total of 41% of the household were food secure, while only 10.5% were facing severe food insecurity.

### Rate of stunting and underweight and PoB

The present study found a substantial percentage of children with undernutrition (stunting-35.8% and underweight-27.1%) (Table 2). We observed a relatively higher rate of stunting (52.7%) and underweight (41.1%) in children with birth order > 3 and < 2 years of IBBSB, followed by birth order 2–3 children and < 2 years of IBBSB (stunting-41.5% and underweight-32.3%). The association between the birth order of children and the prevalence of undernutrition was highly significant.

**Table 2:** Rate of stunting and underweight by predictors

Variables	Stunting (%)	95% CI	***p***-value	Underweight (%)	95% CI	***p***-value
**Total**	**35.8**	**33.4,38.3**		**27.1**	**24.7,29.7**	
**Planning of births**						
Order 1 & < 12 month of IBMFB	36.7	29.6,44.5	*p* = 0.000	19.1	13.7,25.9	*p* = 0.000
Order 1 & 12–24 months of IBMFB	28.6	24.0,33.6		24.6	20.0,29.9	
Order 1 & 25–36 months of IBMFB	30.7	23.3,39.3		23.5	16.2,32.8	
Order 1 & > 36 months of IBMFB	25.0	18.2,33.3		16.9	10.9,25.3	
Order 2–3 & < =24 months of IBBSB	41.5	35.2,48.2		32.3	26.2,39.0	
Order 2–3 & > 24 months of IBBSB	34.1	30.5,38.0		24.7	21.2,28.5	
Order > 3 & < =24 months of IBBSB	52.7	41.5,63.5		41.1	30.9,52.1	
Order > 3 & > 24 months of IBBSB	46.8	40.0,53.6		40.4	33.2,47.9	
**Mother’s age at marriage in years**						
< 15	42.4	36.4,48.6	*p* = 0.001	33.4	27.7,39.7	*p* = 0.009
15–19	37.5	34.5,40.5		28	25.4,30.7	
20–24	27.0	23.1,31.2		21.4	17.0,26.5	
25–40	32.4	20.3,47.5		21.2	12.3,34.1	
**Mother’s current age in years**						
15–19	37.8	29.6,46.8	*P* = 0.022	25.6	19.3,33.3	*p* = 0.052
20–24	31.6	28.1,35.2		22.9	19.8,26.2	
25–29	34.9	30.4,39.7		29.5	25.2,34.1	
30–34	42.1	36.2,48.2		30.4	25.2,36.2	
35–49	42.6	34.8,50.7		30.3	23.4,38.3	
**Mother’s BMI**						
Normal	36.2	33.5,38.9	*p* = 0.000	26.4	23.6,29.4	*p* = 0.000
Underweight	44.5	38.6,50.5		42.7	36.8,48.9	
Obese	23.8	18.8,29.7		10.7	7.6,14.9	
**Mother’s anemia**						
Not anemic	35.7	32.4,39.0	*p* = 0.927	24.9	21.9,28.0	*p* = 0.017
Anemic	35.9	32.6,39.3		29.7	26.5,33.1	
**Caste**						
Brahmin/Chettri	34.7	31.1,38.6	*p* = 0.044	24.1	21.0,27.5	*p* = 0.000
Terai/Madhesi other	41.9	36.3,47.6		37.1	32.2,42.4	
Dalit	39.1	32.6,46.0		30.8	24.5,37.8	
Newar/Janajatis	31.7	27.2,36.5		20.0	16.0,24.6	
Muslim/other	34.7	27.2,43.0		33.8	21.8,48.4	
**Mother’s education**						
Illiterate	45.8	41.9,49.8	*p* = 0.000	36.8	32.5,41.3	*p* = 0.000
Primary	36.7	32.2,41.5		27.9	23.7,32.5	
Secondary	30.2	26.9,33.7		21.4	18.2,24.9	
Higher	22.4	17.6,28.1		14.8	11.0,19.7	
**Father’s education**						
Illiterate	46.3	39.9,52.8	*p* = 0.000	38.1	32.3,44.3	*p* = 0.000
Primary	40.3	35.6,45.2		30.2	25.3,35.7	
Secondary	33.9	30.7,37.3		24.8	21.8,28.0	
Higher	25.3	20.6,30.7		19.6	15.4,24.7	
**Mother’s education**						
Not working	30.0	26.5,33.8	*p* = 0.000	24.9	21.2,29.0	*p* = 0.054
Agriculture	40.6	37.2,44.0		30.2	26.9,33.6	
Paid jobs	36.8	30.5,43.5		23.3	17.9,29.8	
**Wealth quintile**						
Poorest	49.1	44.2,54.1	*p* = 0.000	33.0	28.2,38.3	*p* = 0.000
Poorer	38.3	33.6,43.2		27.8	23.3,32.8	
Middle	35.4	30.3,40.9		32.8	27.4,38.5	
Richer	32.4	27.6,37.5		23.6	19.2,28.6	
Richest	17.6	13.2,23.0		12.9	8.7,18.7	
**Exposure to mass media (newspaper/radio/television)**						
Not at all	50.5	45.7,55.3	*p* = 0.000	39.7	34.0,45.7	*p* = 0.000
Less than once a week	32.1	28.9,35.4		22.9	20.1,26.1	
At least once a week	30.4	27.0,33.9		24.0	20.5,27.9	
**Sex of the children**						
Male	35.9	32.8,39.1	*p* = 0.953	27.0	24.2,30.0	*p* = 0.921
Female	35.8	32.4,39.3		27.2	24.0,30.8	
**Age of the children**						
0–11 months	16.4	12.7,20.8	*p* = 0.000	18.5	14.8,23.0	*P* = 0.001
12–23 months	37.7	32.7,43.0		28.9	24.2,34.1	
24–35 months	44.8	40.0,49.7		31.1	26.5,36.0	
36–47 months	39.8	35.0,44.8		26.5	22.3,31.2	
48–59 months	40.8	35.3,46.4		30.7	25.8,36.1	
**Place of residence**						
Urban	31.9	28.7,35.3	*p* = 0.001	23.6	20.7,26.9	*p* = 0.005
Rural	40.2	36.6,44.0		31.0	27.1,35.1	
**Ecological region**						
Mountain	46.8	38.8,54.9	*p* = 0.011	28.2	21.2,36.6	*p* = 0.000
Hill	32.7	29.1,36.6		18.8	15.9,22.0	
Terai	36.5	33.2,40.0		32.4	29.0,36.0	
**Province**						
Province 1	32.7	27.1,38.8	*p* = 0.001	24.4	18.4,31.5	*p* = 0.000
Province 2	36.6	32.8,40.6		36.6	31.6,42.0	
Province 3	30.7	23.9,38.5		14.5	9.2,22.1	
Province 4	29.0	22.1,37.0		15.4	10.6,22.0	
Province 5	38.0	31.0,45.5		27.2	21.9,33.3	
Province 6	54.9	48.6,61.1		36.4	30.8,42.4	
Province 7	35.4	29.4,42.0		27.2	22.8,32.1	
**Household food security**						
Food secure	29.7	26.2,33.5	*p* = 0.000	22.4	19.1,26.2	*p* = 0.003
Mild food insecure	35.7	31.4,40.2		27.4	23.4,31.9	
Moderate food insecure	41.4	36.7,46.2		31.1	26.9,35.6	
Severe food insecure	46.4	38.3,54.7		34.9	26.5,44.4	

Mothers who were in the age group 35–49 at the time of the survey had the highest prevalence of child stunting (42.6%) and showed a significant association. Children born to the mothers in the age category of 30–34 years had the highest prevalence of underweight (30.4%) but did not show significant association. Mother’s age at marriage less than 15 years (stunted-42.4% and underweight-33.4%), underweight women (stunted-44.5% and underweight-42.7%), mother’s illiteracy (stunting-45.8% and underweight-36.8%), father’s illiteracy (stunting-46.3% and underweight-38.1%), poorest wealth quintile (stunted-49.1% and underweight-33.0%), no exposure to mass media (stunted-50.5% and underweight-39.7%), children’s age group 24–35 months (stunted 44.8% and underweight-31.1%), and rural residence (stunted-40.2% and underweight-31.0%) were the significant predictors of childhood undernutrition.

### Factors associated with stunting

Multiple logistic regression showed that children with birth order 1 and 12–24 months of IBMFB had 0.6 times significantly lower odds of being stunted than children with birth order one and < 12 months of IBMFB (Table 3). None of the other birth orders and birth intervals showed a significant association with stunting (Table 3). Mothers in the age group 20–24 years had significantly lower odds of having stunted children than mothers in the age group < 15 years. Obese mothers had decreased odds of child stunting than mothers with normal BMI. The relationship was statistically significant. However, the relationship between underweight mothers and child stunting was not significant. Mothers with paid jobs had significantly higher odds of stunted children than mothers who were not working. All categories of wealth quintile (poorer, middle, richer, and richest) had significantly lower odds of child stunting than those who were in the poorest quintile. Exposure to mass media also had significantly decreased odds of stunting than those who did not have exposure at all. The odds ratio was 0.6 for those who were exposed less than once a week. For those who were exposed at least once a week, the odds ratio was again 0.6. We observed highly significant greater odds of stunting in all age categories of the children than the 0–11 months age category. At the administrative level, Province 6 had greater odds of stunting than Province 1.

**Table 3:** Results from logistic regression analysis for the likelihood of stunting in underfive children

Variables	Adjusted Odds ratio	95% CI
Order 1 & < 12 month of IBMFB	Reference group	
Order 1 & 12–24 months of IBMFB	0.6*	0.4–0.9
Order 1 & 25–36 months of IBMFB	0.7	0.4–1.2
Order 1 & > 36 months of IBMFB	0.6	0.3–1.0
Order 2–3 & < =24 months of IBBSB	0.9	0.6–1.4
Order 2–3 & > 24 months of IBBSB	0.8	0.5–1.1
Order > 3 & < =24 months of IBBSB	1.3	0.6–2.5
Order > 3 & > 24 months of IBBSB	0.9	0.5–1.6
**Mother’s age at marriage in years**		
< 15	Reference group	
15–19	1.1	0.8–1.4
20–24	0.8	0.5–1.3
25–40	1.3	0.6–2.8
**Mother’s current age in years**		
15–19	Reference group	
20–24	0.6*	0.4–0.9
25–29	0.6	0.4–1.1
30–34	0.8	0.4–1.6
35–49	0.6	0.3–1.3
**Mother’s BMI**		
Normal	Reference group	
Underweight	1.3	1.0–1.7
Obese	0.6*	0.4–0.9
**Mother’s anemia**		
Not anemic	Reference group	
Anemic	1.0	0.8–1.3
**Caste**		
Brahmin/Chettri	Reference group	
Terai/Madhesi other	1.3	0.8–2.1
Dalit	1.0	0.6–1.5
Newar/Janajatis	0.9	0.6–1.2
Muslim/other	0.9	0.6–1.5
**Mother’s education**	Reference group	
Illiterate	0.8	0.6–1.1
Primary	0.9	0.7–1.3
Secondary	0.9	0.5–1.4
Higher	0.8	0.6–1.1
**Father’s education**		
Illiterate	Reference group	
Primary	1.0	0.7–1.5
Secondary	1.0	0.7–1.4
Higher	0.9	0.6–1.6
**Mother’s occupation**		
Not working	Reference group	
Agriculture	1.1	0.9–1.4
Paid jobs	1.7**	1.2–2.4
**Wealth quintile**		
Poorest	Reference group	
Poorer	0.7*	0.5–0.9
Middle	0.6**	0.4–0.8
Richer	0.5**	0.3–0.8
Richest	0.3***	0.2–0.6
**Exposure to mass media (newspaper/radio/television)**		
Not at all	Reference group	
Less than once a week	0.6**	0.5–0.9
At least once a week	0.6***	0.5–0.8
**Sex of the children**		
Male	Reference group	
Female	0.9	0.7–1.1
**Age of the children**		
0–11 months	Reference group	
12–23 months	3.2***	2.2–4.6
24–35 months	4.8***	3.3–7.1
36–47 months	3.7***	2.4–5.6
48–59 months	3.8***	2.5–5.8
**Place of residence**		
Urban	Reference group	
Rural	1.1	0.9–1.4
**Ecological region**		
Mountain	Reference group	
Hill	0.8	0.5–1.2
Terai	1.1	0.7–1.8
**Province**		
Province 1	Reference group	
Province 2	0.7	0.4–1.0
Province 3	1.1	0.7–1.6
Province 4	1.1	0.7–1.8
Province 5	1.1	0.7–1.6
Province 6	1.8**	1.1–2.7
Province 7	0.8	0.5–1.2
**Household food security**		
Food secure	Reference group	
Mild food insecure	1.0	0.7–1.3
Moderate food insecure	1.1	0.8–1.5
Severe food insecure	1.2	0.8–1.8

*** *p* < 0.001, ** *p* < 0.01, * *p* < 0.05

### Factors associated with underweight

Table 4 presents the results of the logistic regression analysis for the odds of underweight in under-five children. Any category of PoB did not show a statistically significant relationship with underweight than first-order birth and less than 12 months of IBMFB. Underweight mothers were 1.7 times more likely to have underweight children than mothers with normal BMI. In contrast, obese mothers had significantly lower odds of underweight children than mothers with normal BMI. Poorer, richer, and richest category of wealth quintile showed significantly lower odds of underweight children than the poorest quintile. We also observed significantly higher odds of underweight children in the age group 12–23 months, 24–35 months, and 48–59 months than children in the 0–11 months category. At ecological division, the Terai region had 1.8 times higher odds of underweight children than the mountain region. The relationship was statistically significant.

**Table 4:** Results from logistic regression analysis for the likelihood of underweight in underfive children

Variables	Odds ratio	95% CI
Order 1 & < 12 month of IBMFB	Reference group	
Order 1 & 12–24 months of IBMFB	1.3	0.8–2.1
Order 1 & 25–36 months of IBMFB	1.2	0.6–2.2
Order 1 & > 36 months of IBMFB	0.9	0.5–1.7
Order 2–3 & < =24 months of IBBSB	1.2	0.7–2.1
Order 2–3 & > 24 months of IBBSB	1.1	0.7–1.7
Order > 3 & < =24 months of IBBSB	1.4	0.7–2.7
**Mother’s age at marriage in years**		
< 15	Reference group	
15–19	0.9	0.7–1.3
20–24	0.9	0.6–1.6
25+	1.4	0.6–3.4
**Mother’s current age in years**		
15–19	Reference group	
20–24	1.0	0.6–1.5
25–29	1.3	0.8–2.3
30–34	1.4	0.8–2.6
35+	1.2	0.6–2.5
**Mother’s BMI**		
Normal	Reference group	
Underweight	1.7***	1.3–2.2
Obese	0.4***	0.3–0.7
**Mother’s anemia**		
Not anemic	Reference group	
Anemic	1.0	0.8–1.2
**Caste**		
Brahmin/Chettri	Reference group	
Terai/Madhesi other	1.0	0.7–1.6
Dalit	1.0	0.6–1.4
Newar/Janajatis	0.8	0.5–1.1
Muslim/other	0.8	0.4–1.5
**Mother’s education**		
Illiterate	Reference group	
Primary	0.9	0.6–1.2
Secondary	1.0	0.7–1.4
Higher	0.7	0.4–1.2
	0.9	0.6–1.2
**Father’s education**		
Illiterate	Reference group	
Primary	1.0	0.7–1.5
Secondary	1.0	0.7–1.4
Higher	1.0	0.6–1.7
**Mother’s education**		
Not working	Reference group	
Agriculture	1.2	0.9–1.6
Paid jobs	1.3	0.9–2.0
**Wealth quintile**		
Poorest	Reference group	
Poorer	0.6*	0.4–0.9
Middle	0.7	0.4–1.1
Richer	0.5**	0.3–0.8
Richest	0.4**	0.2–0.8
**Exposure to mass media (newspaper/radio/television)**		
Not at all	Reference group	
Less than once a week	0.8	0.5–1.0
At least once a week	0.7	0.5–1.0
**Sex of the children**		
Male	Reference group	
Female	1.0	0.8–1.2
**Age of the children**		
0–11 months	Reference group	
12–23 months	1.6**	1.2–2.2
24–35 months	1.9***	1.3–2.7
36–47 months	1.4	0.9–2.0
48–59 months	1.9**	1.3–2.7
**Place of residence**		
Urban	Reference group	
Rural	1.1	0.9–1.5
**Ecological region**		
Mountain	Reference group	
Hill	0.9	0.6–1.4
Terai	1.8*	1.1–3.0
**Province**		
Province 1	Reference group	
Province 2	1.0	0.6–1.7
Province 3	0.7	0.5–1.2
Province 4	0.8	0.5–1.4
Province 5	1.0	0.6–1.5
Province 6	1.5	0.9–2.3
Province 7	0.9	0.6–1.3
**Household food security**		
Food secure	Reference group	
Mild food insecure	0.9	0.6–1.2
Moderate food insecure	1.0	0.7–1.4
Severe food insecure	1.2	0.7–1.8

*** *p* < 0.001, ** *p* < 0.01, * *p* < 0.05

### Interaction of PoB with maternal nutrition and wealth quintile

We tested for the interaction effect of PoB with maternal anemia for both stunting and underweight separately (not shown in the table). We also examined the interaction between PoB and household wealth quintile. We did not observe any significant interaction.

## Discussion

In the present study, we found that the rate of stunting and underweight in children under 5 years of age increased significantly with an increase in birth order and shorter birth interval. However, the overall result of multivariate logistic regression did not specifically show a significant relationship between the PoB and undernutrition. We found that younger age at marriage, poor socioeconomic characteristics of the mother, area of residence were significantly associated with childhood undernutrition.

### Rate of stunting and underweight and PoB

The stunting rate and underweight rate of children under 5 years of age in Nepal are quite similar to the national stunting rate and underweight rate in children under 5 years of selected South Asian countries. Pakistan DHS 2017/18 reported an overall stunting rate of 37.6% and an underweight rate of 23% [28]. The stunting rate and underweight rate in Bangladesh were 31 and 22%, respectively, as per Bangladesh DHS 2017/18 [29]. In contrast, the prevalence rate of stunting and underweight in children under 5 years of age in the Maldives is relatively less than the prevalence rate in other South Asian countries. Maldives DHS 2016/17 reported an only national prevalence rate of 15.3% stunting and 15% underweight [30]. The continuous decline in the prevalence of childhood undernutrition in Nepal since 2001 can be attributed to the rigorous implementation of several large-scale nutrition-specific projects and programs led by GoN, with the help of development partners.

This study showed that the prevalence of stunting and underweight increases with higher birth order and shorter birth intervals. This finding was consistent with previous studies that sought to examine the relationship between PoB and childhood undernutrition. For example, a retrospective analysis of the National Family Health Survey (NFHS) 2015/16 data of India also reported the highest rate of stunting (53.1%) and underweight (48.4%) in children with birth order > 3 and < 3 years of IBBSB [15]. Rana et al. found a lower risk for stunting (20%; *p* < 0.01) and underweight (14%; *p* < 0.05) in the first birth order with > 24 months of IBMFB in their retrospective study that analyzed DHS data of selected South Asia countries (Nepal, India, Bangladesh, and Pakistan) [20]. A retrospective study analyzing a large-scale survey in India to investigate the relationship between birth interval and childhood undernutrition also reported a 28% increase in stunting and 26% increase in underweight for those children born with a birth interval of < 24 months [25].

### Factors associated with stunting and underweight

This study showed significantly lower odds of stunting only in children with birth order one and 12–24 months of IBMFB than those children with birth order one and < 12 months of IBMFB. However, the present study did not yield a significant relationship between PoB and underweight. The result of a retrospective study analyzing the 2002–2003 El Salvador NFHS data to examine the relationship between birth spacing and childhood undernutrition also showed significantly higher odds of stunting in birth intervals of < 24 months (OR 1.52, 95% CI 1.21–1.92) and intervals of 25–35 months (OR 1.30, 95% CI 1.05–1.64) than intervals of 36–59 months [23]. As in the present study, this study also did not show a significant relationship between birth interval and underweight. Peter et al. reported birth interval as a significant predictor of child stunting in a cross-sectional study that aimed to measure the prevalence and identify the predictors of undernutrition in children age 0–59 months in Hyderabad, India (OR 1.82, 95% CI 1.03–3.21) [31].

The result of the multivariate logistic regression in the present study showed that low BMI was not significantly associated with stunting. However, low BMI mothers had significantly higher odds of underweight children than mothers with normal BMI. In contrast, a cross-sectional study examining the factors associated with child stunting and underweight in 35 low-and middle-income countries showed significantly increased odds of child stunting (OR 1.6, 95% CI 1.6–1.7, *p* < 0.001) in mothers with low BMI [32]. A significant association was also observed for underweight (OR 2.3, 95% CI 2.1–2.4, *p* < 0.001) [32]. Nahar et al., in their case-control study, also reported that severely underweight children were more likely to have underweight mothers (AOR 3.8, 95% CI 2.6–5.4) [33]. A cross-sectional study conducted in ten slums of Hyderabad, India, to appraise caregiving practices and health and nutritional status of children under 5 years of age reported low BMI of mother to be the significant predictor of stunting (OR 1.99, 95% CI 1.5–4.7) [31]. Another retrospective study by Yang et al. reported significantly decreased odds of stunting for obese mothers (OR 0.70, 95% CI 0.61–0.79, *p* < 0.001), which was consistent with the outcome of the present study (OR 0.6, 95% CI 0.4–0.9, *p* < 0.05) [34].

Mother’s in the age group 20–24 years had a significantly lower odds of stunting than mothers in the age group 15–19 years in the present study. Another case-control study in Bangladesh that investigated the risk factors associated with severe underweight among young children aged 6–24 months also reported the mother’s age < 19 years as a strong risk factor to cause child underweight (AOR 3.0, 95% CI 1.9–4.8) [33]. In contrast, Yang et al. reported significantly higher odds of stunting in the age group 20–30 (OR 1.18, 95% CI 1.08–1.29, *p* < 0.001) [34].

We obtained significantly lower odds of stunting and underweight in poorer, middle, richer, and the richest wealth quintile than those who were in the poorest wealth quintile. Li et al. also reported that the poorest household wealth was the strongest factor associated with both child stunting (OR 1.7, 95% CI 1.6–1.8, *p* < 0.001) and underweight (OR 1.2, 95% CI 1.1–1.3, *p* < 0.001) [32]. A retrospective study analyzing Uganda DHS to examine determinants of stunting in children under 5 years of age reported similar higher odds of stunting in the poorest wealth quintile (OR 1.73, 95% CI1.45–2.06, *p* ≤ 0.001) [34]. Another retrospective study analyzing NDHS data also reported higher odds of stunting among babies born to poorer families than those born to wealthier families (AOR 1.51, 95% CI 1.23–1.87) [35].

The present study showed that children > 11 months are a strong predictor of stunting and underweight. A community-based cross-sectional study that examined factors associated with underweight among children under 5 years of age in Eastern Nepal reported that children who were > 24 months of age were more likely to be underweight than children < 24 months (OR 2.72, 95% CI 1.6–4.7) [36].

To our knowledge, this is one of the few studies in Nepal to examine the relationship between PoB and childhood undernutrition. However, this study has a few known limitations. We used the NDHS 2016 cross-sectional survey data, but a causal relationship between the associated factors and outcome cannot be inferred from this study. There is a possibility of residual confounding.

## Conclusion

The findings of the present study inferred that planning of birth has a protective effect on childhood undernutrition. PoB can increase the length of time between the marriage and first birth, birth intervals between the subsequent births, and can also limit the number of childbirths, thus enabling mothers to have an ample amount of time to recover physically and emotionally. Hence, mothers can improve their health and nutrition, which ultimately contributes to children’s better nutritional status. In a country like Nepal, where 25% of the population lives below the absolute poverty line [37], a focus to uplift the economic status of these populations is quintessential to enable them to practice PoB as a strategy to mitigate childhood undernutrition. The wide geographical coverage and utilization of mass media across the country can also play a crucial role in informing and educating people on PoB and its importance to reduce childhood undernutrition. To sum up, this study provides some statistical evidence, which government authorities, plans and policymakers can use to advocate PoB strategies (integration of nutritional strategies with PoB interventions) to fight against childhood undernutrition. The government of Nepal can use the findings of this study as supportive evidence to formulate and execute evidence-based nutrition-related policies, plans, and programs.

## Data Availability

The datasets used and/or analyzed in the current study are available from the DHS program (http://www.dhsprogram.com/data/available-datasets.cfm) on reasonable request.

## Abbreviations

AARR: Annual Average Rate of Reduction
AOR: Adjusted Odds Ratio
BMI: Body Mass Index
CAPI: Computer Assisted Personal Interview
CBS: Central Bureau of Statistics
CI: Confidence Interval
DoHS: Department of Health Service
DHS: Demographic and Health Survey
GoN: Government of Nepal
IBBSB: Interval Between Birth and Subsequent Birth
IBMFB: Interval Between Marriage and First Birth
IRB: Institutional Review Board
IYCF: Infant and Young Children Feeding
KISAN: Knowledge-based Integrated Sustainable Agriculture in Nepal
OR: Odds Ratio
MoHP: Ministry of Health and Population
MSNP: Multi-Sector Nutrition Plan
NDHS: Nepal Demographic and Health Survey
NFHS: Nepal Family Health Survey
NHRC: Nepal Health Research Council
NPHC: Nepal Population and Housing Census
PoB: Planning of Birth
SABAL: Sustainable Action for Resilience and Food Security
SD: Standard Deviation
SUN: Scaling Up Nutrition
UN: United Nation
UNICEF: United Nations Children’s Fund
USAID: United States Agency for International Development
WHA: World Health Assembly
WHO: World Health Organization
